# Construction of a Band‐Aid Like Cardiac Patch for Myocardial Infarction with Controllable H_2_S Release

**DOI:** 10.1002/advs.202204509

**Published:** 2022-10-26

**Authors:** Weirun Li, Peier Chen, Yuxuan Pan, Ling Lu, Xiaodong Ning, Jiamin Liu, Jintao Wei, Minsheng Chen, Peng Zhao, Caiwen Ou

**Affiliations:** ^1^ Affiliated Dongguan Hospital Southern Medical University (Dongguan People's Hospital) Dongguan 523058 China; ^2^ Department of Cardiology Laboratory of Heart Center Heart Center Zhujiang Hospital Southern Medical University Guangzhou 510280 China; ^3^ NMPA Key Laboratory for Research and Evaluation of Drug Metabolism Guangdong Provincial Key Laboratory of New Drug Screening School of Pharmaceutical Sciences Southern Medical University Guangzhou 510515 China; ^4^ Guangdong Provincial Key Laboratory of Cardiac Function and Microcirculation Southern Medical University Guangzhou 510515 China; ^5^ Guangdong Provincial Key Laboratory of Shock and Microcirculation Guangzhou 510515 China

**Keywords:** myocardial infarction, hydrogen sulfide, cardiac patch, macrophage, reactive oxygen

## Abstract

Excessive or persistent inflammation incites cardiomyocytes necrosis by generating reactive oxygen species in myocardial infarction (MI). Hydrogen sulfide (H_2_S), a gaseous signal molecule, can quickly permeate cells and tissues, growing concerned for its cardioprotective effects. However, short resident time and strong side effects greatly restrict its application. Herein, a complex scaffold (AAB) is first developed to slowly release H_2_S for myocardial protection by integrating alginate modified with 2‐aminopyridine‐5‐thiocarboxamide (H_2_S donor) into albumin electrospun fibers. Next, a band‐aid like patch is constructed based on AAB (center) and nanocomposite scaffold which comprises albumin scaffold and black phosphorus nanosheets (BPNSs). With near‐infrared laser (808 nm), thermal energy generated by BPNSs can locally change the molecular structure of fibrous scaffold, thereby attaching patch to the myocardium. In this study, it is also demonstrated that AAB can enhance regenerative M2 macrophage and attenuate inflammatory polarization of macrophages via reduction in intracellular ROS. Eventually, this engineered cardiac patch can relieve inflammation and promote angiogenesis after MI, and thereby recover heart function, providing a promising therapeutic strategy for MI treatment.

## Graphical abstract

A band‐aid like patch based on albumin electrospun scaffold is developed, which can in situ release H_2_S in a controllable way and adhere to the surface of the heart under NIR light irradiation. The H_2_S‐release patch presents a promising therapeutic strategy to repair injured myocardial tissue.

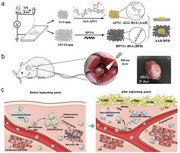

## 1 Introduction

Myocardial infarction (MI) is the most common cause of heart failure (HF). It is always caused by prolonged restriction of blood flow to myocardium and can lead to necrosis of cardiomyocytes.^[^ ^1^, ^2^ ^]^ Currently, strategy for HF treatment has changed from short‐term correction of hemodynamic parameters to long‐term rehabilitation approaches and therapy of HF has made great progress. ^[^ ^3^ ^]^ However, curative effects for HF caused by myocardial damage and necrosis are not very good. HF is still associated with high mortality and hospital readmission rates.^[^ ^4^ ^]^ With the progression of HF, the only clinically effective treatment for end‐stage HF is heart transplant which is limited by the insufficient matching hearts supply. Therefore, the therapeutic strategies that enable to reduce the scar tissue and recover heart functions are necessary to prevent HF.^[^ ^5^ ^]^ The repair of necrotic myocardium needs to take into account many factors, such as oxidative stress, ischemia and hypoxia, inflammation, and so on.^[^ ^6^, ^7^, ^8^ ^]^

As endogenous gas signaling molecule, hydrogen sulfide (H_2_S) plays a critical role in the protection against oxidative stress, inflammation and apoptosis in cardiovascular diseases including hypertension, myocardial ischemia and HF.^[^ ^9^, ^10^, ^11^ ^]^ H_2_S can affect injured cells through complex mechanism, one of which is decreasing generation of reactive oxygen species (ROS) and the regulation of phenotypic polarization of macrophages.^[^ ^12^, ^13^ ^]^ Although several H_2_S donors (e.g., ATB‐346, GIC‐1001 and GYY4137) have been explored in clinical trials,^[^ ^14^ ^]^ the therapeutic effectiveness can't be assured due to its toxicity, poor water solubility and high clearance rates.^[^ ^15^, ^16^ ^]^ Currently, the administration of H_2_S in biomedical research is mainly the injection of inorganic sulfide salt or H_2_S donor‐loaded modified hydrogen in situ.^[^ ^17^, ^18^, ^19^ ^]^ However, man‐made damage caused by intramuscular injection and unclear side effects of synthetic macromolecular polymer greatly limit the development and application of H_2_S.

In recent years, electrospun fiber scaffold has attracted great interests in heart regeneration for its extracellular matrix (ECM)‐mimicking structure, which can provide appropriate mechanical support and promote cardiomyocyte assembly into functional tissues.^[^ ^20^, ^21^, ^22^ ^]^ The capability of absorbing nanoparticles and molecules through covalent or noncovalent forces also endow them with extended functions.^[^ ^23^ ^]^ It is regarded to be a promising therapy strategy to fabricate a functional cardiac patch through combination of electrospinning techniques and natural proteins (e.g., serum albumin) in the last decade.^[^ ^24^, ^25^ ^]^ Serum albumin serves as an appropriate electrospun fiber substrate because of its easy availability and well biocompatibility.^[^ ^26^, ^27^, ^28^, ^29^ ^]^ Some studies have shown that serum albumin can be used as protein solder for laser tissue welding.^[^ ^30^, ^31^, ^32^, ^33^, ^34^ ^]^ Basing on this, KM McNally et al. developed an ICG‐doped albumin protein solder to repair bovine aorta specimens.^[^ ^35^ ^]^ Lauto, A. et al. designed an albumin‐genipin compound material for sheep intestine healing.^[^ ^32^ ^]^ Besides, Malki et al. prepared a nanocomposite scaffold by integrating gold nanorods into albumin electrospun membrane, which is strongly attached to the surface of the heart after near‐infrared (NIR) laser (808 nm) irradiation without suture or addition of chemical adhesive.^[^ ^36^ ^]^ Considering that, it will be meaningful to construct electrospun fiber scaffolds with controlled H_2_S release ability and systematically study how it works in the process of myocardial tissue repair.

The hypoxia‐ischemia condition and blocked electrical signal transmission are not conducive to myocardium repair process.^[^ ^37^, ^38^, ^39^ ^]^ Thus, stimulating angiogenesis and increasing intercellular signal transduction will be beneficial to recover heart functions. As an outstanding 2D layered nanomaterial, black phosphorus nanosheets (BPNSs) contribute a lot to the treatment of various diseases for its great biocompatibility, electrical conductivity and NIR photothermal conversion.^[^ ^40^, ^41^ ^]^ Some studies suggested that black phosphorus can not only promote angiogenesis but also foster the process of neurogenesis after ischemic injuries.^[^ ^42^, ^43^, ^44^, ^45^ ^]^ Combining with the composite scaffolds developed by Malki et al., we hypothesized that the BPNSs‐loaded electrospun albumin scaffolds can adhere to the heart under the irradiation of NIR and help recover heart function with fine biocompatibility and medical value.

**Scheme 1:**
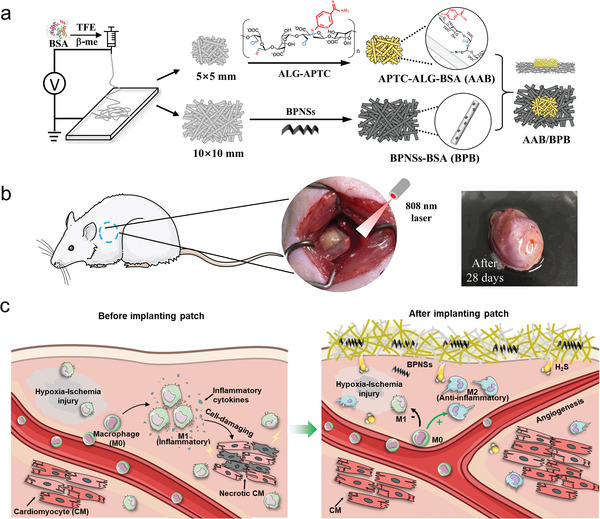
Schematic illustration of the construction of the band‐aid like cardiac patch (AAB/BPB), and its application in the treatment of myocardial infarction. a) The preparation process of the patch; b) the implantation of AAB/BPB; c) underlying mechanism of the therapeutic effects of AAB/BPB.)

In this study, we propose to improve heart function and reverse cardiac remodeling through implanting a H_2_S and BPNSs releasing band‐aid like cardiac patch onto infarcted area. The patch is equipped with two modules (**Scheme** **1**), the first module is the 2‐aminopyridine‐5‐thiocarboxamide (APTC) functional albumin scaffold (APTC‐ALG‐BSA, AAB), the second module is a BPNSs loaded albumin scaffold (BPB). As an organic thiol‐dependent H_2_S donors, APTC had been widely explored in clinical trials for its relatively release ability.^[^ ^14^, ^46^ ^]^ The AAB/BPB patch can tightly adhere to the myocardial tissues after irradiated by NIR. A homogenizing change in protein with interdigitation of altered individual fibrils appears to be the structural basis of the welding effect.^[^ ^31^, ^33^, ^47^ ^]^ Besides, the increased temperature at the patch during laser treatment can denature the albumin. It can change or destroy its secondary and tertiary spatial structure, and increase viscosity by making the hydrophobic groups originally inside the molecules expose to the surface.^[^ ^48^, ^49^ ^]^ The results in vitro and in vivo indicate that the band‐like AAB/BPB patch can regulate the phenotypic transformation of macrophages and promote vessels creation, which help recover the rat's heart function. These findings support that the construction of electrospun albumin scaffolds with multiple functions to improve repairing conditions after MI holds great potentials for cardiac tissue engineering.

**Figure 1:**
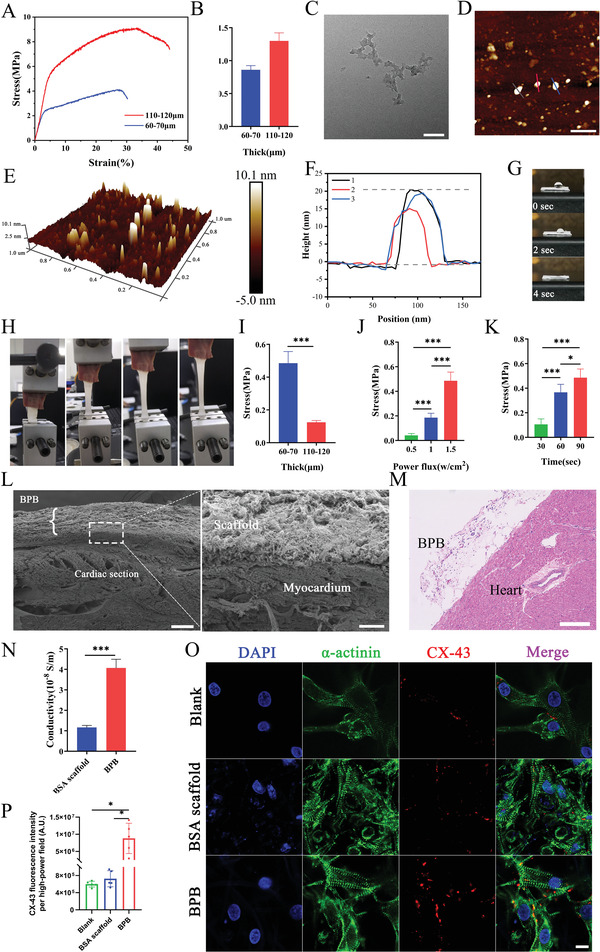
The characterization of functional electrospun fiber scaffold. A) Representative stress–strain graph and B) Young's modulus of BSA scaffolds with different thickness (60–70 µm and 110–120 µm) (*n* = 3). C) TEM image of black phosphorus nanoplates (BPNSs). Scale bar: 100 nm. D,E) AFM image of BPNSs. Scale bar: 200 nm. F) AFM measured thickness of BPNSs. G) Digital pictures of water droplets placed on BSA scaffolds. H) Evaluation of tissue adhesion by a mechanical tester with a porcine cardiac tissue. I) Stress of BPB with different thicknesses attached on the myocardium (90 s, at 1.5 W cm^−2^) (*n* = 5). Stress of BPB (60–70 µm) attached on the myocardium with J) different NIR illumination power fluxes (90 s) or K) different NIR illumination times (at 1.5 W cm^−2^) (*n* = 5). L) SEM images of the cross‐section between BPB and myocardium. Scale bar: 150 and 15 µm. M) Hematoxylin–eosin (HE) staining of heart tissue 28 days after treatment. Scale bar: 200 µm. N) Conductivities of different scaffolds (*n* = 5). O) Immunofluorescent staining of connexin 43 (CX‐43) (scale bar: 10 µm), and its statistical analysis (P) (*n* = 5). Quantified data are presented as presented as means ± S.E., and significance was evaluated via Student's *t* test. *p < 0.05, **p < 0.01, ***p < 0.001.

## 2 Results

### 2.1 The Adhesive Strength and Conductivity of the Engineered Cardiac Patch

Bovine serum albumin (BSA) was utilized as a substrate to prepare electrospun protein scaffold, then the functional scaffolds AAB and BPB were separately constructed and integrated under mild pressure. The mechanical properties of BSA scaffold with different thickness was examined. **Figure** **1**A shows the representative stress–strain profiles of different scaffolds. The stress has a good linear relationship with the strain in a certain range and the Young's Modulus value of different scaffolds can be calculated from the stress–strain curve. The value of the Young's Modulus of BSA scaffolds (0.86 ± 0.06 MPa) with a thickness of 60–70 µm was a bit closer to that of the native heart ECM (0.36 ± 0.1 MPa) than that 110–120 µm (Figure 1B).^[^ ^27^ ^]^ The cell experiments also prove that the BSA scaffold matches the contractile function of cardiomyocytes (Movies S1 and S2, Supporting Information). Besides, the water was completely absorbed into the BSA scaffold after 4 s, indicating that the scaffolds have good hydrophilic surface (Figure 1G).

The BPNSs with a diameter of (143.3 ± 70.2) nm (Figure 1C) were synthesized by liquid stripping method, in which the black phosphorus (400 µg mL^−1^) solution was dispersed under ultrasound for 7 h. The average thickness of BPNSs was (18.1 ± 2.3) nm (Figure 1D‐F). The BPB can be constructed by evenly adding BPNSs solution (20 µL, 200 µg mL^−1^) onto the BSA scaffold (10 mm × 10 mm). A mechanical tester was used to evaluate the tissue adhesion of BPB with a porcine cardiac tissue (Figure 1H). The adhesion of BPB with different thickness to tissue after NIR light (808 nm) irradiation (at 1.5 W cm^−2^) for 90 s was tested. The scaffold of 60–70 µm attaches greatly stronger to the tissues compared to the 110–120 µm scaffold (Figure 1I), probably because of better penetration of the light. As the strong adhesion of scaffold is based on the structural changes of albumin molecules caused by the photothermal effect of BPNSs, the irradiation condition of NIR light was detailly optimized. As shown in Figure 1J,K, the adhesive strength of BPB enhances with the increase of near‐infrared laser power (0.5, 1.0, and 1.5 W cm^−2^) and the irradiation time (30, 60, and 90 s). This may be resulted from that both power increasing and time prolonging will raise local temperature of BPB (Figure S1, Supporting Information), however, too fast heating rate or high temperature can be accompanied by a high risk of myocardial injury. To obtain optimal tissue adhesion and prevent potential damage to myocardium caused by excessive temperature, the BPB with a thickness of 60–70 µm under NIR irradiation (1.5 W cm^−2^) for 60 s was used in the subsequent experiments.

In order to further investigate the attachment of BPB scaffold to tissues, animal experiment was carried out. The rat heart was taken out on day 28 after operation (Scheme 1). Observed by SEM, BPB scaffold tightly adhered to the myocardium, revealing that BPB scaffolds can effectively attach to the surface of the heart after irradiation (Figure 1L). The heart section with hematoxylin and eosin staining further verified the integration of patch and tissue (Figure 1M).

BPNSs has not only a high photothermal conversion efficiency but also excellent electromechanical properties.^[^ ^42^ ^]^ We find BPB has a better electrical conductivity than BSA scaffold after adding BPNSs (Figure 1N and Figure S2, Supporting Information). The results in vitro experiment show connexin 43 protein (CX‐43) expression significantly enhanced in primary myocardial cells grown in BPB compared with the other groups (Figure 1O,P). That further suggests addition of conducting material BPNSs probably profit transmission of cardiac electrical signal and provide a favorable microenvironment for tissue repair.

### 2.2 The Controlled H_2_S Release of AAB Scaffold

**Figure 2:**
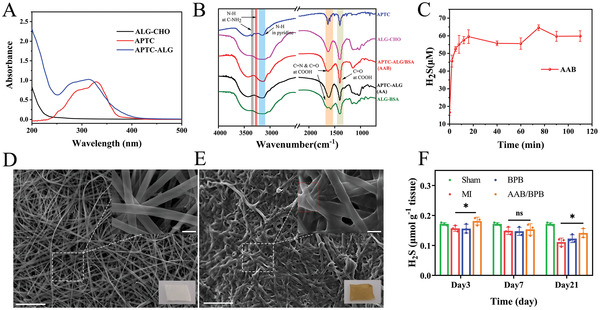
The characterization of APTC‐ALG‐BSA scaffold (AAB). A) UV–visible absorption spectra wave scan of ALG‐CHO, APTC and AA. B) FTIR spectra of BSA scaffold, APTC, ALG‐CHO, APTC‐ALG (AA), ALG‐BSA (AB), and AAB. C) H_2_S‐releasing profile in vitro (*n* = 3). D,E) SEM images of BSA scaffold and AAB. Scale bar: 50 µm and 5 µm (inset Figure). F) Sulfide concentration of the cardiac tissue in the MI area (*n* = 3). The data are shown as mean values ± SD, *p* values are based on Student's *t* test. *p < 0.05, **p < 0.01, ***p < 0.001.

In order to construct AAB, the alginate was firstly oxidized by sodium periodate (NaIO_4_) to produce active aldehyde groups. As the degradation rate of alginate is strongly inhibited with the oxidation,^[^ ^50^ ^]^ (64.4 ± 4.7)% of degree of oxidation was selected to obtain sufficient aldehyde groups with better in vivo degradation performance (Table S1, Supporting Information). Next, Schiff base reaction between the aldehyde groups of oxide alginate (ALG‐CHO) and amino groups of APTC were employed to develop a functional copolymer APTC‐ALG (AA) (Figure S3, Supporting Information). AA was characterized by ^1^H NMR spectrum, C‐NMR spectrum and UV–visible absorption spectra. Single peak at 8.38 ppm is attributed to hydrogen atom adjacent to nitrogen atom on the pyridine ring that exists only in APTC (Figure S4, Supporting Information). Peaks appeared between 3.50 ppm and 3.65 ppm relate to partially oxidized alginate. Additionally, the result of C‐NMR spectrum further verified the successful combination of AA for the peaks above 90 ppm which existed only APTC (Figure S5, Supporting Information). The absorption from 250 to 400 nm of AA in the **Figure** **2**A is also resulted from the specific structure of APTC. The mass fractions of APTC in AA are calculated as 2.33 wt% based on the content of N element (Table S2, Supporting Information). Next, AAB can be acquired by the Schiff base reaction between surplus aldehyde groups of AA and amino groups of BSA scaffold (5 mm × 5 mm).

As shown in the Fourier transform infrared spectrometry (FTIR) spectra of different materials (Figure 2B), the peak at 3350 cm^−1^ (*ν*_N–H_) of APTC is disappeared, indicating the occurrence of Schiff base reaction between APTC and AA. Compared to ALG‐CHO, distinctive wide and notched absorption peak of AAB and ALG‐BSA appearing near the 1630 cm^−1^ can be attributed to the overlap of carbon–nitrogen double bond in the structure of the Schiff‐based and carbon–oxygen double bond in substrate. It can be seen from scanning electronic microscopy (SEM) that the albumin scaffold is made up of smooth fibers with a width of about 4 µm (Figure 2D), and the fibers of AAB scaffold is covered with a layer of flocs which is in agreement with the microstructure of alginate (Figure 2E).

The H_2_S‐releasing rate of AAB was determined by the methylene blue assay (Figure 2C). AAB shows a sustained and controlled release of H_2_S when triggered by reduced glutathione, also indicating the successful combination of AA and BSA scaffold. Next, the total sulfide concentration was detected by using the methylene blue method to indirectly reflect H_2_S concentration in the infarction on day 3, 7, 21 after coronary artery ligation. Endogenous H_2_S can signal through some cardioprotective pathways to maintain cardiovascular homeostasis and impaired production of H_2_S is associated with the pathology of cardiovascular disease, such as coronary artery disease and hypertension.^[^ ^10^, ^51^, ^52^, ^53^ ^]^ Myocardial injury induced by sustained ischemia can consume a lot of endogenous H_2_S, which lead to a decrease in H_2_S production and indued more myocyte apoptosis.^[^ ^54^, ^55^ ^]^ That is why the total H_2_S concentration of cardiac tissue in the sham group is higher than that of in the MI group including nontreated or treated with AAB/BPB (Figure 2F). On day 3, the concentration of H_2_S in myocardial infarct area in AAB/BPB group is detected as (0.181 ± 0.013) µmol g^−1^, which is higher than that in MI group (0.156 ± 0.010) µmol g^−1^ and BPB group (0.155 ± 0.015) µmol g^−1^. The result suggests that the AAB can release H_2_S controllably within a few days after MI. Besides, from Figure 2F, we can know that the concentration of hydrogen sulfide in infarct zone on day 21 in AAB/BPB group (0.141 ± 0.015) µmol g^−1^ is higher than MI group (0.111 ± 0.013) µmol g^−1^, which may be related to high survival rate of cardiomyocytes. In the following experiments, we will further explore how composite scaffold protect cardiomyocyte from ischemia injure.

### 2.3 Biocompatibility of the Functional Scaffolds

**Figure 3:**
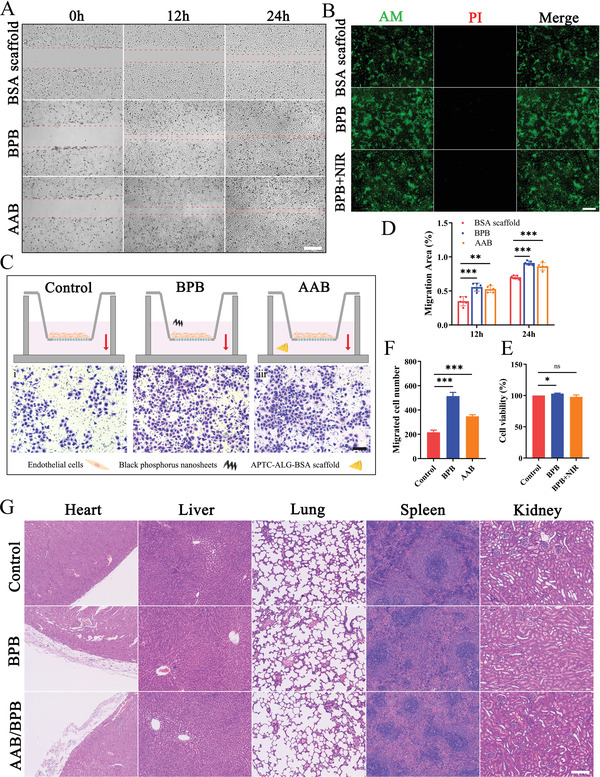
Biological toxicity of BPB and AAB and their effects on HUVECs migration. A) The cell scratch test for different treatments different time (0, 12, 24 h). Scale bar: 200 µm. B) Representative images of neonatal rat primary cardiomyocytes stained with Calcein‐AM (green) and PI (red), which were seeded on the scaffold. Scale bar: 400 µm. C) Transwell assay. Scale bar: 150 µm. D) Quantitative analysis of migration area shown in figure A (*n* = 5). (E) Cell viability of cardiomyocytes presented in figure B (*n* = 3). F) Quantitative evaluation of the Transwell assay presented in figure C (*n* = 5). G) HE staining of heart, liver, lung, spleen and kidney 28 days after intervention. Scale bar: 200 µm. All data are presented as the mean values ± SD, and significance was evaluated via Student's *t* test. *p < 0.05, **p <0.01, ***p <0.001.

The migration of endothelial cells plays a key role in the process of tissue repair and regeneration.^[^ ^56^, ^57^, ^58^ ^]^ Thus, the effects of BPB and AAB on the migration of human umbilical vein endothelial cells (HUVECs) were also explored in the scratch wound model as well as Transwell assay. The HUVECs migrated evidently in BPB group and AAB group compared to the BSA scaffold group (**Figure** **3**A). It is also noted that BPB can significantly promote HUVECs migration compared to AAB. The cell migration area can reach 91.06% and 86.11% after 12 h scratch in BPB group and AAB group (Figure 3D). This was further proved according to the result of Transwell assay (Figure 3C,F). The robust promotion of BPNSs on the endothelial cell migration may be resulted from that the BPNSs can promote the proliferation of endothelial cells and higher electrical conductivity of it help enhance cell‐to‐cell connections.^[^ ^59^, ^60^ ^]^ Collectively, both BPB and AAB have a well biosafety and a migration boost on endothelial cells, which is the core of angiogenesis.

Live/dead staining assay was then used to examine the cytotoxicity of the AAB and BPB with or without NIR irradiation. The cell morphologies in BPB without or with NIR irradiation for 60 s at 1.5 W cm^−2^ are similar to BSA scaffold group (Figure 3B). Statistical analysis indicates that there is no significant difference in cell survival rate between control group and BPB with irradiation group (Figure 3E). This is due to good biocompatibility of phosphate, which is a main degradation product of BPNSs.^[^ ^61^, ^62^, ^63^ ^]^ The fluorogram and statistical analysis in Figure S6 (Supporting Information) shows that AAB has no obvious toxicity to cardiomyocytes compared with the blank and BSA scaffold group. In vivo, there were no obviously pathological structure or cell morphology changes of major organs of rats (Figure 3G; Figure S7C–E, Supporting Information) on day 28 after implantation. In addition, immunohistochemical staining of IL‐1*β* indicates that the patch does not cause significant inflammatory response to myocardium (Figure S7A,B, Supporting Information). In a word, AAB/BPB patch possesses a better biocompatibility and bioactivity.

### 2.4 AAB Induces M2 Macrophage Polarization and Inhibits Inflammatory Macrophage in Vitro

**Figure 4:**
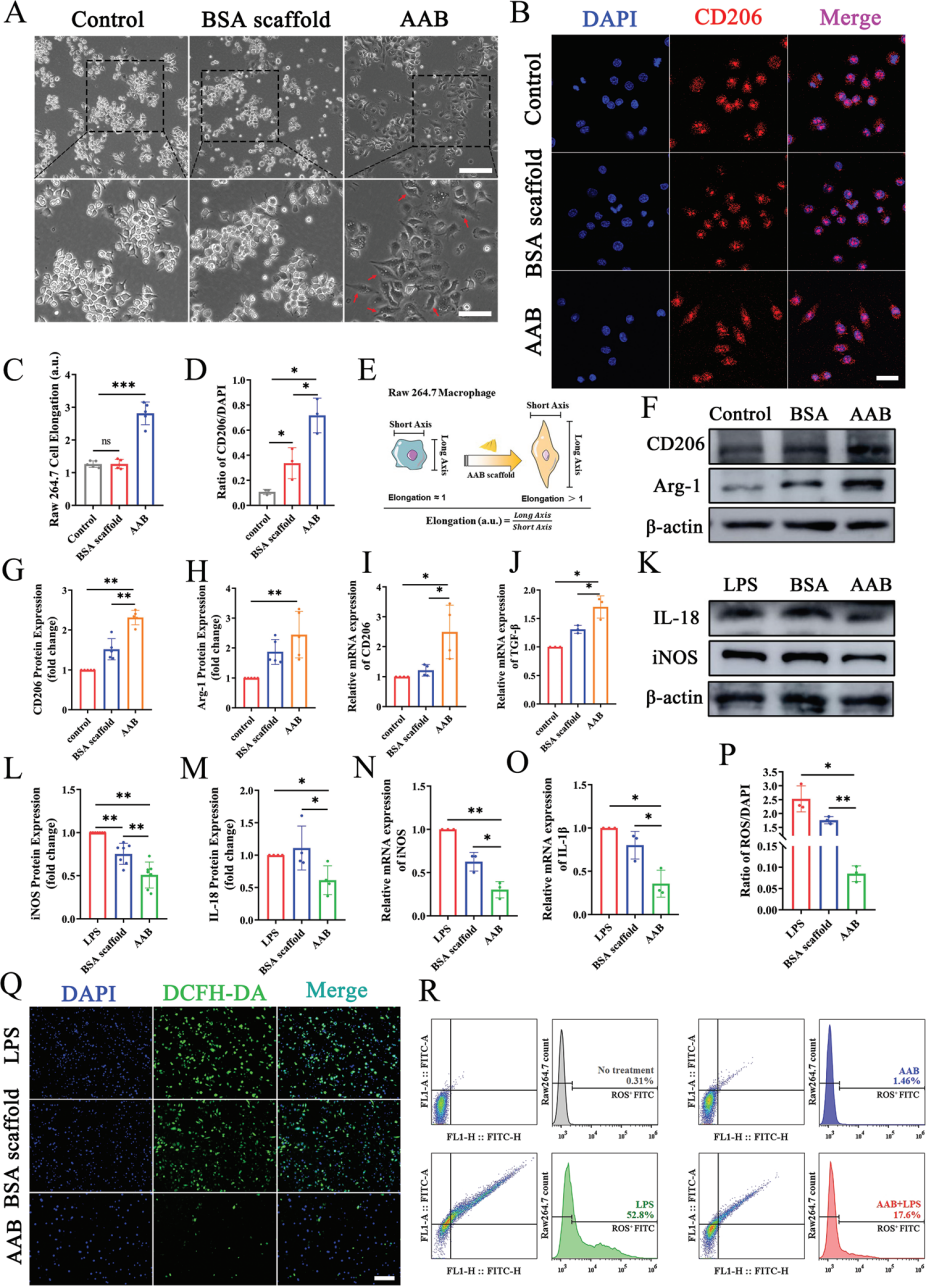
AAB treatment induced the polarization of Raw 264.7 macrophages to M2 phenotype and inhibited inflammatory macrophages in vitro. A) Cell morphological change of macrophages after 24 h incubation with AAB observed by optical microscope. Red arrow indicated elongated macrophages. Scale bar: 100 µm (above) and 50 µm (below). B) Representative images of CD206 immunofluorescence staining of macrophages. Scale bar: 25 µm. C) Statistical data of the cell elongation change (*n* = 5). D) Quantitative analysis of CD206 expression in figure B (*n* = 3). E) The schematic of the cell morphological change affected by AAB treatment (Cell elongation was defined as a ratio of long axis to short axis length). F) M2 macrophage related protein (CD206 and Arg‐1) expression level conducted by western blotting. G,H) Quantification of western blotting bands (*n* = 5). I,J) Relative mRNA expression of CD206 (*n* = 4) and TGF‐*β* (*n* = 3). K) M1 macrophage related protein expression level of iNOS and IL‐18 conducted by western blotting in macrophages. L,M) Quantification of western blotting bands (iNOS, *n* = 7; IL‐18, *n* = 4). N,O) Relative mRNA expression of M1 macrophage surface marker iNOS and IL‐1*β* in different groups (*n* = 3). P) Quantitative analysis of intracellular ROS (*n* = 3). Q) Representative images of DCFH‐DA fluorescent staining of ROS in macrophages under different treatments for 24 h. Scale bar: 200 µm. R) Flow cytometric analysis of DCFH‐DA fluorescent labeled macrophages. All data are shown as mean values ± SD, *p* values are based Student's *t* test. *p <0.05, **p <0.01, ***p <0.001; ns, nonsignificant.

Considering that the driving macrophage differentiation toward M2 phenotype may be one of mechanism of anti‐inflammation of H_2_S.^[^ ^64^, ^65^, ^66^ ^]^ Here, we hypothesized that AAB scaffold can exert its cardioprotective effect through the transformation of macrophage phenotype in myocardial tissue. Raw 264.7 macrophages were used as a model target, which were incubated with AAB scaffold for 24 h, and then morphologies were observed under an optical microscope. As seen in **Figure** **4**A, macrophages cultured in medium without any treatment or with BSA scaffold appears morphologically round and clustered. While loosely compact cells and more macrophages with elongated shape which are marked with red arrowheads in magnified image can be observed in the AAB treatment group. The elongation of macrophages is defined as a ratio of long axis length to short axis length (Figure 4E). The control group and BSA scaffold group displays elongation of (1.26 ± 0.10) a.u. and (1.26 ± 0.14) a.u., while the AAB group exhibited that of (2.82 ± 0.35) a.u., 2.2 times higher than the control (Figure 4C). Immunofluorescence staining of CD206, a surface marker of M2 macrophages, was performed to further confirm the M2 phenotype polarization of AAB. After the exposure to AAB scaffold for 24 h, Raw 264.7 macrophages stretched out to the sides presenting pseudopod‐like structures, in contrast to the round shape of the control group and BSA scaffold group (Figure 4B). Statistically, elongated macrophages treated with AAB scaffold had higher ratio of CD206/DAPI (Figure 4D), indicating the polarization state of macrophage is related to M2 phenotype.^[^ ^67^ ^]^ Western blot and qRT‐PCR analysis were also applied to further confirm the effects of AAB on macrophage polarization (Figure 4F). After incubation with AAB for 24 h, the expression of M2 macrophage‐related protein, including CD206 and Arg‐1, were increased compared to control group (Figure 4G,H). The results of qRT‐PCR analysis also verify that incubation with AAB can enhance the expression of CD206 and TGF‐*β*, which are the typical M2 macrophage‐related genes (Figure 4I,J). These results suggest that H_2_S released from AAB can induce the macrophage polarization to M2 anti‐inflammatory type with elongated cell morphology and enhance expression of M2 phenotype maker.

Next, to evaluate the effect of AAB on inflammatory macrophage (M1), lipopolysaccharide (LPS) was used induce Raw264.7 macrophage polarization toward M1 macrophage.^[^ ^68^, ^69^, ^70^, ^71^ ^]^ Raw264.7 macrophages were treated with different scaffolds following the addition of culture medium containing 500 ng mL^−1^ LPS. The expression of inflammation‐related proteins and genes were also verified by western blot and qRT‐PCR analysis. The incubation with AAB can significantly decrease the expression of iNOS and IL‐18 (Figure 4K‐N), which are the two biomarkers of inflammatory M1 macrophage.^[^ ^72^, ^73^ ^]^ It suggests that AAB can exert anti‐inflammatory effect, further proven by the decrease of expression of inflammation‐related gene IL‐1*β* (Figure 4O).

Infarction‐ or ischemia‐mediated production of reactive oxygen species (ROS) plays a crucial role in triggering an inflammatory response including recruiting inflammatory cells to the infarct and promoting M1 differentiation.^[^ ^7^, ^71^, ^74^ ^]^ H_2_S can exert its anti‐inflammatory effect by travelling through cell membranes in a free permeation process.^[^ ^75^, ^76^ ^]^ Therefore, we speculated that the mechanism responsible for AAB‐mediated macrophage polarization may be related with the scavenging of ROS by H_2_S. After 24 h LPS‐treatment, the level of ROS was detected through DCFH‐DA fluorescence kit and further analyzed with fluorescence microscope (Figure 4Q). In the absence of LPS, the ROS‐positive cell counts are very low in each group (Figure S8, Supporting Information). Macrophages treated with LPS present stronger green fluorescence than that without LPS. The green fluorescence intensity in AAB group decreases significantly compared to that in the LPS group and BSA scaffold group (Figure 4P), demonstrating the potential eliminated capability of ROS by H_2_S released from AAB scaffold.

Next, fluorescence‐activated cell sorting (FACS) was applied to further evaluate the production of ROS within the macrophages (Figure 4R). Approximately 0.31% and 52.8% of Raw264.7 macrophages without or with LPS treatments present ROS‐positive green FITC fluorescence, respectively. The AAB group following LPS treatment dramatically reduces FITC‐positive cell counts to 17.6%, further confirming the ROS scavenging of H_2_S through free diffusion after stimulation of LPS and achieving anti‐inflammatory effect. Besides, when macrophages were treated with BSA scaffold alone, about 12.7% of Raw264.7 macrophages showed ROS‐positive green FITC fluorescence (Figure S9, Supporting Information). The above results indicate that the reduce of ROS within macrophages is caused by AAB instead of BSA scaffold.

With the results mentioned above, we can know that AAB induce M0 macrophages to M2 reparative macrophages and inhibit generation of M1 inflammatory macrophages through scavenging of ROS. What's more, from Figure 4L,N, we find that BSA scaffold may promote generation of M1 inflammatory macrophages. On one hand, albumin fiber can induce immune response due to different species sources of albumin and macrophages, which is verified by the increased intracellular ROS in BSA scaffold group (Figure S9, Supporting Information). And potential adsorption properties of BSA scaffold for LPS may be responsible for lower expression of iNOS, contrasted with LPS group. On the other hand, specific markers selected for distinguishing M2 and M1 macrophages may not be detected merely in corresponding phenotypes. These two macrophage phenotypes represent the opposite ends of the polarization and the phenotypic transition between M1 and M2 is continuous, which means macrophages in transition state can express both M1 and M2 markers. Thus, lower increased protein makers (CD206 and Arg‐1) expression can be seen in BSA scaffold group.

### 2.5 AAB/BPB Patch Regulates Infiltrated Macrophages and Promotes Angiogenesis in Infarcted Myocardium

**Figure 5:**
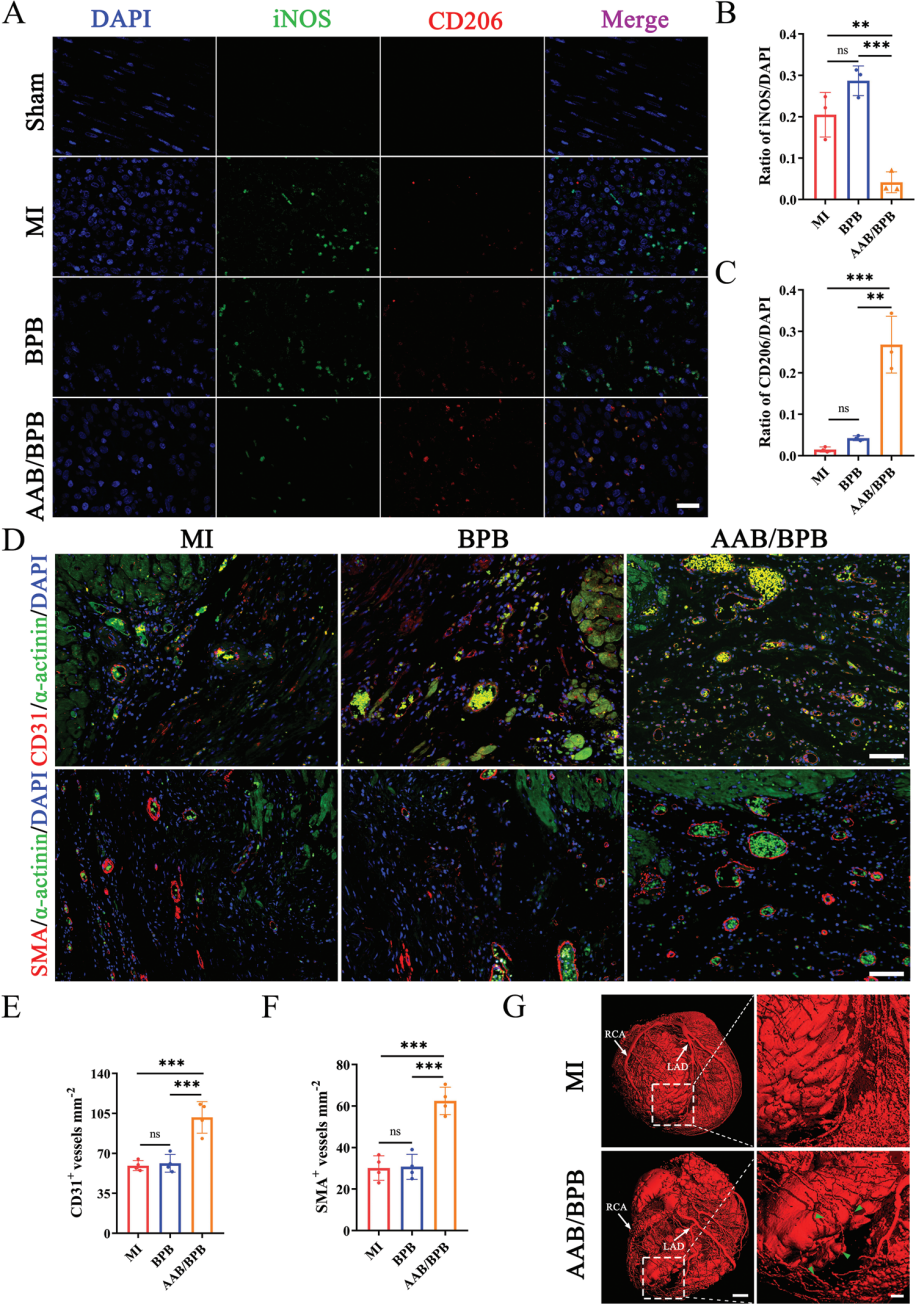
Evaluation of AAB/BPB patch on macrophages polarization at infarct area 4 days post treatment and angiogenesis inducement 28 days after MI. A) Representative images of CD206 (red) and iNOS (green) immunofluorescence staining of the sham group, MI group, BPB and AAB/BPB group. Scar bar: 25 µm. B) and C) Quantitative analysis of iNOS and CD206 expression in macrophages (*n* = 3). D–F) Sections from the border zone of the infarction were collected at day 28 and immunofluorescent staining for the endothelial marker CD31 and *α*‐smooth muscle actin (SMA). D) Cardiomyocytes were visualized by staining for *α*‐actinin, and nuclei were counterstained with DAPI. Scale bar: 75 µm. Border zone vascular density E) and arterial density F) were quantified as the number of vessel‐like structures that express CD31 and SMA, respectively (*n* = 4). G) Images of micro‐CT scanning after injecting the prepared Microfil contrast agent. Scale bar: 2 mm and 500 µm. Quantified data are presented as means ± SE, and *p* value are based on one‐way ANOVA. *p < 0.05, **p < 0.01, ***p <0.001; ns, nonsignificant.

Previous studies indicated that circulating monocytes infiltrated into tissue and differentiated into inflammatory macrophages at day 2 after myocardial ischemic injury.^[^ ^77^, ^78^ ^]^ Subsequently, transition from inflammatory phase to reparative phase happened between 3 and 5 days after infarction.^[^ ^79^ ^]^ To examine the effect on infiltrated macrophages, an MI rat mode was established through ligation of the left anterior descending (LAD) coronary arteries, then AAB/BPB patch was planted on the left ventricular epicardial surface following the NIR irradiation. The cardiomyocytes of rat heart were harvested for immunofluorescent staining on day 4 after MI (**Figure** **5**A).^[^ ^71^ ^]^ iNOS and CD206 were applied to present the infiltration of inflammatory and anti‐inflammatory macrophages, respectively.^[^ ^80^, ^81^, ^82^, ^83^ ^]^ Fluorescent images shows that many iNOS‐positive cells (green) accumulate in the infarct area in MI group and BPB group, while CD206‐positive cells are rarely observed (Figure 5A,B). In contrast, CD206‐positive cells were increased in the infarct with the treatment of AAB/BPB scaffold, which is verified by a higher ratio of CD206/DAPI compared to the other two groups (Figure 5C). The results suggest that the treatment of composite AAB/BPB patch can achieve the targeting resolution of inflammation by facilitating effective transition of the inflammatory phase to the reparative phase to enhance the population of M2 macrophages.

The results mentioned above (Figure 3) indicate that both BPNSs and AAB can promote endothelial cells migration which is a very important initiating process for angiogenesis. Therefore, 4 weeks after MI, the blood vessels in myocardium were observed by fluorescence microscopy (Figure 5D). Vascular and arterial density remained significantly higher in the border zone of AAB/BPB‐treated hearts than that of BPB‐treated or nontreated hearts (Figure 5E,F). However, there was no obvious difference between MI group and BPB group. Besides, with the 3D vascular reconstruction by micro‐CT scanning, we can find that the treatment of AAB/BPB patch can help the establishment of collateral circulation (as pointed by the green triangle) and thereby enhance improvement of ischemic myocardial perfusion (Figure 5G).

### 2.6 Evaluation of Heart Functions and Left Ventricular Remodeling

**Figure 6:**
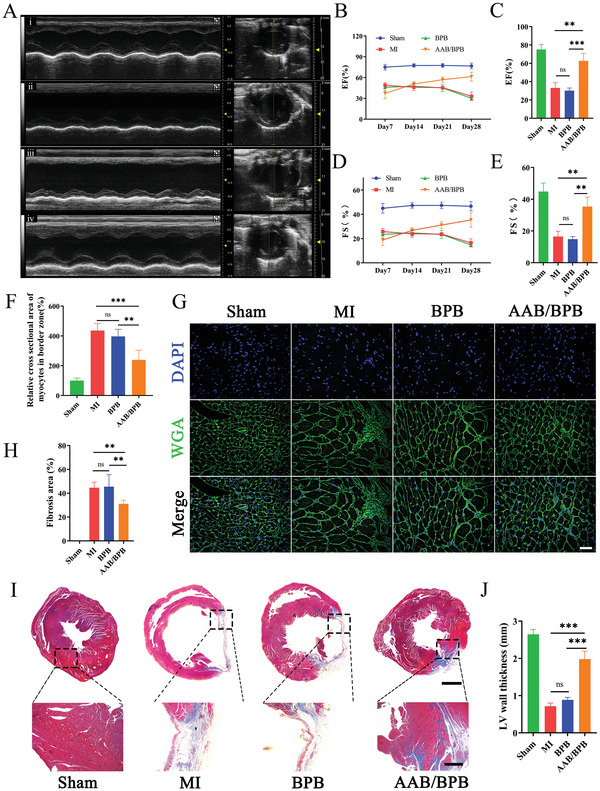
The cardiac functions and left ventricular remodeling of rats were assessed 28 days postoperatively. A) Representative echocardiography (ECHO) images of different groups after 28 days (i, Sham; ii, MI; iii, BPB; iv, AAB/BPB). Echocardiographic assessments of left ventricular ejection fraction (EF) B) and fractional shortening (FS) D) were conducted at 7, 14, 21, and 28 days after MI and treatment (*n* = 3). Statistical analysis of EF C) and FS E) on day 28 (*n* = 3). Sections from the infarction were collected at day 28 and cardiomyocytes membrane were visualized by staining for wheat germ agglutinin (WGA) G). Scale bar: 25 µm. F) Quantitative analysis of relative cross‐sectional area of myocytes (*n* = 4). H) Fibrosis area based on I) (*n* = 4). I) Masson's‐trichrome staining for collagen (blue) and muscle (red). Scale bar: 2 mm and 150 µm. J) Left ventricular wall thickness based on (I) (*n* = 4). Quantified data are presented as means ± SD, and significance was evaluated via One‐way ANOVA. **p <0.01, ***p <0.001; ns, nonsignificant.

Benefiting from its strong tissue adhesion, angiogenesis and efficient promotion to anti‐inflammatory macrophages, the therapeutic effects of AAB/BPB patch on acute myocardial infarction were investigated. Echocardiography was performed to assess left ventricle (LV) functions of rat heart on 7, 14, 21, and 28 days after the surgery (**Figure** **6**A). The ejection fraction (EF) and fractional shortening (FS) of rats in MI and BPB group dropped continuously with the time extension, while a gradually enhancement can be observed in AAB/BPB group (Figure 6B,D). Four weeks after interventions, AAB/BPB‐treated rats had a higher EF and FS than the others in general (Figure 6C,E). What's more, AAB/BPB treatment decreases the end‐diastolic volume (EDV) and end‐systolic volume (ESV), obviously different from the other interventions (Figure S10, Supporting Information). Additionally, the results of continuous echocardiography in normal rats with or without AAB/BPB prove that the therapeutic patch doesn't affect normal cardiac function (Figure S11, Supporting Information). Periodic ECG detections indicate that BPB or AAB/BPB do not cause obvious disturbance of electrical signal conduction (Figure S12, Supporting Information). The rats were sacrificed at 4 weeks postoperatively. Partial degradation of the patch can be observed on the heart (Figure S13, Supporting Information).

Improving heart function and reversing cardiac remodeling are the key to decrease the incidence of heart failure. Following ischemic injury, increased apoptosis of the cardiomyocytes and significant matrix remodeling cause cardiac fibrosis.^[^ ^84^, ^85^ ^]^ LV remodeling then happens with local myocardial wall thinning and progressive ventricular dilation. Cardiomyocyte hypertrophy is also an important pathological changes of LV remodeling. In this study, wheat‐germ agglutinin (WGA) was applied to observe the morphological changes of cardiomyocytes in border zone (Figure 6G). Myocardial cells in border zone hypertrophied 28 days after injury, (4.36 ± 0.47) times bigger than that in the sham group (Figure 6F). To note is that AAB/BPB treatment significantly inhibited myocardial hypertrophy compared with the other treatment, suggesting AAB/BPB can help prevent LV remodeling. Next, Masson's trichrome staining was further employed to determine the protective effects of AAB/BPB on decreasing cardiac fibrosis. As shown in Figure 6I, the blue collagen deposition area is obvious in the MI group 4 weeks after surgery, which indicates a severe fibrosis of the myocardium. After treatment of AAB/BPB patch, the fibrotic area of MI hearts decreases sharply compared to the other two groups (Figure 6H). A thinning ventricular wall is observed in the MI group as well as BPB group but it becomes more mitigatory in the AAB/BPB group. According to the quantitative data, the fibrosis area decreases from (44.6 ± 4.8) % to (31.0 ± 3.2) %, and the left ventricular wall thickness increases from (0.7 ± 0.1) mm to (2.0 ± 0.2) mm compared to the MI group (Figure 6H,J). These results demonstrate that the AAB/BPB scaffold can reduce the LV cavity size, restore LV geometry and improve LV functions. The remarkable recovery of cardiac geometry and function can be attributed to the synergetic effect of black phosphorus nanosheets and H_2_S from ABB/BPB electrospun scaffold together with NIR irradiation enhanced adhesion.

## 3 Discussion

APTC is a thiol‐dependent H_2_S donor,^[^ ^46^ ^]^ however, poor hydrophilicity and high clearance rates limit its further application. In this study, we developed a band‐aid like cardiac patch based on albumin electrospun fiber scaffold, which can in situ release H_2_S in a controllable way and adhere to the surface of the heart without suture or mixing with synthetic adhesive. It will greatly minimize the required dosage of the H_2_S donor compared to the systemic H_2_S administration. The patch was consisting of two parts, one was a H_2_S‐releasing scaffold (AAB, 5 mm × 5 mm) which was placed in the center, and another was a BPNSs loading scaffold (10 mm × 10 mm). AAB was constructed by integrating alginate modified with APTC into BSA electrospun fibers. Finally, the band‐aid like cardiac patch was positioned on the myocardium and irradiated with a near IR laser (808 nm). The BPNSs were able to absorb the light and converted it to thermal energy, which locally changed the molecular structure of the albumin fibrous scaffold and promote the attachment to the wall of the heart. This may be caused by melting the polymer, or by denaturation of albumin and collagen upon heating and their interlock.^[^ ^30^, ^47^ ^]^ The structural and biochemical character of albumin, such as hydrophobic cavities and negative charge, allowed its application as a protein carrier for enhancing the pharmacokinetic profile of protein‐ or peptide‐based drugs.^[^ ^86^, ^87^ ^]^ Previous research suggested electrospun scaffolds made with albumin can support the assembly of functional cardiac tissue that generate strong contraction forces.^[^ ^27^ ^]^ More importantly, unlike the scaffold prepared by Malki et al.,^[^ ^36^ ^]^ we used BPNSs to generate thermal energy instead of gold nanoparticles. Catabolite produced by BPNSs was phosphate ions, suggesting that the BPNSs had a better biocompatibility than nano gold.^[^ ^41^, ^61^ ^]^ Besides, the results show that the addition of BPNSs can enhance the conductivity of the scaffold and help signal transduction of cardiomyocytes in vitro.

Stimulated by thiol compounds in the tissue, such as cysteine, GSH, regulatory protein with ‐SH and so on,^[^ ^88^ ^]^ AAB then slowly released H_2_S to exert cardioprotective effect through phenotypic modulation of macrophages in myocardial infarct. Unlike direct exposure to the reaction system in vitro, it is a gradual process that sulfhydryl compounds seep from myocardial tissue to be adsorbed by albumin scaffold on the surface of the heart. Therefore, the contact reaction between AAB and sulfhydryl substances may occur after implantation for a longer time and achieve a sustained H_2_S release rate in vivo, being demonstrated by the higher concentration of sulfide in infarct zone in AAB/BPB‐treated heart than that in nontreated or BPB‐treated heart on day 3 after operation. Inflammatory macrophages can accumulate in the infarct zone for several days after MI,^[^ ^89^, ^90^ ^]^ which is served as a “double‐edged sword” as the early inflammation is required for tissue repair. However, prolonged inflammation will be adverse to proper tissue reconstruction.^[^ ^82^, ^91^ ^]^ Mounting evidences indicate that M2 macrophages play a vital role in infarct repair and an early shift from the inflammatory M1 phase to the reparative M2 phase is beneficial to improvements of cardiac function after MI.^[^ ^82^, ^83^, ^92^, ^93^ ^]^ The alteration of macrophages morphology has contact with their functional polarization states and elongated macrophages preferred to polarize into M2 macrophages.^[^ ^94^, ^95^ ^]^ By observing the morphological changes and detecting the expression of related phenotypic markers, ^[^ ^96^ ^]^ we find that AAB can promote the polarization of M2 macrophage which exhibited an extended shape, and inhibit inflammatory M1 macrophages through reduction of intracellular ROS in vitro. It is consistent with previous research that H_2_S can transform macrophage phenotype.^[^ ^64^, ^65^, ^66^ ^]^ Although the release of the H_2_S in vivo can only last for several days, it may meet the treatment of myocardial infarction. The infiltration of inflammatory macrophages mainly occurs within days after injury, which suggests that AAB/BPB patch should take effect during this period to save more cardiomyocytes by regulating the inflammation in the infarct. Because the secreted factors from prolonged inflammation, which is widely occurred among patients with MI, will not only further destroy the surviving cardiomyocytes in the infarct, but also harm the healthy cardiomyocytes at the remote.^[^ ^97^ ^]^ The animal experiments further prove that the AAB/BPB patch can timely attenuate inflammation and elicit early polarization toward M2 macrophages, resulting in alleviated cardiac remodeling and promoted heart function recovery. In addition, we also find that the implantation of AAB/BPB help promote vessel creation, which is responsible for the tissue regeneration and repair. But it should be noted that the new blood vessels are mainly induced by AAB/BPB patch based on the results in vitro. There is not enough evidence to support the role of regenerative M2 macrophages on angiogenesis in this article, and more experimental studies are needed in future.

In conclusion, a band‐aid like cardiac patch that can slowly release H_2_S in situ was successfully developed. It can adhere to the heart safely with the aid of NIR. This functional electrospun fiber patch is able to facilitate effective transition of injured myocardium to reparative phase through inducing polarization macrophages to M2 pro‐healing phenotype as well as angiogenesis, presenting a promising therapeutic strategy to repair injured myocardial tissue.

## 4 Experimental Section

### Synthesis and Characterization of ALG‐APTC

Partially oxidized alginate (ALG‐CHO) was prepared following the previous method (Wang et al., 2008). Briefly, 10 g sodium alginate (SA) was solubilized in 50 mL absolute ethanol, and then 8.637 g sodium periodate (NaIO_4_) dissolved in 50 mL deionized water was added under stirring away from light at room temperature. The molar ratio of NaIO_4_ to monomeric unit of SA was 0.8:1. After reaction for 6 h, equimolar ethylene glycol (about 2.23 mL) to NaIO_4_ was added under stirring for 0.5 h to terminate the reaction. Unreacted NaIO_4_ and ethylene glycol was removed by dialysis for a week. A white floccule of ALG‐CHO can be yielded through lyophilization. The APTC‐ALG was synthesized by the Schiff base reaction between aldehyde and amino under mild conditions. First, 0.5 g of ALG‐CHO was dissolved in 5 mL of deionized water, and then 0.049 g of APTC dissolved in 4 mL of DMSO was added dropwise and stirred for 4 h at 50 °C in a nitrogen atmosphere. Afterward, the resultant solution was precipitated with cold ethanol four times to obtain a faint yellow solid of APTC‐ALG. The chemical structure of APTC‐ALG was determined by the UV–vis spectroscopy, ^1^H NMR and Fourier transform infrared spectra.

### Preparation and Characterization of BSA Scaffold and H_2_S‐Releasing Scaffold

1 g bovine serum albumin was dissolved in 5 mL of mixed solution of TFE and distilled water (*W*_TFE_:*W*_water_ = 9:1), and then 134 µL of *β*‐mercaptoethanol was added for overnight reaction. The blend solution was electrospun fiber at room temperature, using a voltage of 12 kV, a spinneret–collector distance of 15 cm, a solution flow rate of 2 mL h^−1^, and an inner needle diameter of 0.4 mm. Next, the electrospun scaffolds of 60–70 µm thick and 110–120 µm thick were collected, dried under vacuum, and kept in a desiccator. The albumin scaffolds were cut into a rectangular shape (25 mm × 10 mm) for tissue‐adhesive test and a square shape (10 mm × 10 mm). Next, 300 mg of APTC‐ALG was dissolved in 1 mL of ultrapure water, then filtered and sterilized. After that, the mixture solution and albumin fiber scaffolds were irradiated with ultraviolet light for 4 h before the scaffolds were soaked in APTC‐ALG solution to react in a shaking table (70 r min^−1^, 37 °C) for another 12 h. Then a faint yellow albumin fiber scaffold with the capability of H_2_S release was obtained.

### H_2_S‐Releasing Profile

H_2_S released from APTC was measured following the methylene blue assay. In short, 5 mg of APTC was dispersed in 50 mL of PBS (pH = 7.4) (0.65 × 10^−3^ m) to form a suspension with uniform particle distribution. And then the suspension was purged vigorously with nitrogen gas for 15 min. Next, 100 mg of GSH was added into the air‐tight flask to ensure a final concentration of 6.5 × 10^−3^ m, triggering the release of hydrogen sulfide gas immediately. At predetermined time points, 250 µL of reaction mixture was withdrawn and added to the mixture of NaOH (1.5 m, 6.25 µL) and zinc acetate (50 µL, 1% w/v in H_2_O), and supernatant was removed after centrifugation at 20500 rcf for 1 h. After that, *N*,*N*‐dimethyl‐*p*‐phenylenediamine sulfate (100 µL, 20 × 10^−3^ m in 7.2 M HCl) and FeCl_3_ (100 µL, 30 × 10^−3^ m in 1.2 m HCl) were added to the precipitation. After dilution with 500 µL of deionized water, the solution was transferred to a 96‐well plate and the optical density was measured at 670 nm after 20 min. Hydrogen sulfide gas released from H_2_S‐releasing scaffold was examined by the same method as described above. Hydrogen sulfide gas released from H_2_S‐releasing scaffold was examined by the same method as described above.

The content of sulfide in myocardial tissue was detected to further evaluate the release of H_2_S in vivo. In brief, after integrating AAB to the heart surface with the help of BPB (as presented in Scheme 1), tissue samples were taken from the myocardial infarction area of the heart after rats anesthetized and sacrificed on 1, 7, 14, and 28 days after MI. And then, to remove residual blood, saline was used to clean the tissue samples. The myocardial infarction tissues were trimmed and weighed with a microbalance and at last homogenized for the methylene blue tests.

### Tissue‐Adhesive Test

The adhesion ability of albumin electrospun scaffold to the surface of the heart was tested using a Lloyd tensile testing instrument with a 50 n load cell at a rate of 5 mm min^−1^. Outer porcine myocardium was cut into pieces with 3 cm × 2 cm size. The scaffolds were placed on the myocardium mass and the overlapping area of the two was set as 1 cm × 1 cm. Following, BPNSs solution (20 µL, 200 µg mL^−1^) was added to the overlapping area and irradiated with ranging laser power fluxes (0.5, 1, 1.5 W cm^−2^) and ranging time (30, 60, 90 s). The 808‐nm diode laser (LR‐MFJ‐808/5000 mW, Changchun, China) was used, and maximal detachment stress of the scaffold from the myocardium was regarded as the adhesive strength (*n* = 5).

### Cell Immunofluorescence

First, Raw 264.7 macrophages were seeded at 6.0 × 10^4^ cells per well plates and cultured for 48 h at 37 °C, and then the medium was replaced by fresh medium (2 mL per well) with the addition of AAB scaffold with 5 mm × 5 mm size for 24 h. Next, medium was carefully removed and cells were washed three times with PBS. Cells were then fixed with 4% paraformaldehyde for 20 min at 37 °C, and permeated with 0.1% Triton X‐100 in PBS for 20 min at 37 °C before being blocked with immunofluorescence blocking solution for 1 h at room temperature. After that, cells were incubated with a rabbit polyclonal antibody to CD206 (1:200, CST) diluted in immunofluorescence primary antibody diluent overnight at 4 °C. Cells were then washed three times with PBS and incubated with a donkey antirabbit IgG Alexa Fluor Cy3‐conjugated secondary antibody (1:500, Bioworld) diluted in immunofluorescence secondary antibody diluent at 37 °C for 2 h in the dark. At last, cells were stained with DAPI to visualize the nuclei. Fluorescence images were captured using a Nikon confocal laser microscope (Japan).

### Western Blot Analysis

The protein expression level of M2 phenotype related protein (CD206 and Arg‐1) and M1 phenotype related protein (iNOS and IL‐18) under different treatments were determined by Western blot analysis. Cell lysates of macrophage were obtained using RIPA lysis buffer supplemented with a cocktail of protease and phosphatase inhibitors on ice for 15 min. After 12 000 × *g* centrifugation at 4 °C, the supernatant was collected to quantify the protein concentration following the bicinchoninic acid (BCA) method. Protein samples were separated by sodium dodecyl sulfate‐polyacrylamide gel (7.5%, 12%) electrophoresis followed by electrophoretic transfer of protein from the gel to a nitrocellulose membrane. The membranes were then blocked with 5% fat free milk in TBST [10 × 10^−3^ m Tris–HCl (pH = 7.6), 100 × 10^−3^ m NaCl and 0.1% Tween 20] for 2 h at room temperature, followed by incubation with primary antibodies at 4 °C overnight. Primary antibodies used here included anti‐CD206, anti‐Arg‐1, anti‐IL18 and anti‐iNOS. Normalization of results was conducted by running parallel Western blots for detecting *β*‐acting protein. The relative intensities of the bands were quantified using an image processing analysis program.

### Quantitative Real‐time Polymerase Chain Reaction

For qRT‐PCR, total RNA was extracted from cultured cells prepared as described before using the TRIzol reagent according to the manufacturer's instructions. The concentration of RNA was tested by a nanodrop 1000 reader (Thermo Scientific). Reverse transcription of total RNA was conducted using a ReverTra Ace‐a kit following the manufacturer's protocol. The primer sequences used for qRT‐PCR were as follows: CD206 (forward: 5′‐CTCTGTTCAGCTATTGGACGC‐3′, reverse: 5′‐TGGCACTCCCAAACATAATTTGA‐3′), TGF‐*β* (forward: TGCTTCAGCTCCACAGAGAA, reverse: TCCAGGCTCCAAATGTAGGG), iNOS (forward: 5′‐ACATCGACCCGTCCACAGTAT‐3′, reverse: 5′‐CAGAGGGGTAGGCTTGTCTC‐3′), and IL‐1*β* (forward: 5′‐AACACAGAAATTATTGTAAAG‐3′, reverse: 5′‐GTCGGAGATTCGTAGCTGGA‐3′). Glyceraldehyde 3‐phosphate dehydrogenase (GAPDH) (forward: 5′‐ CAGTGCCCGAAACCCACAC ‐3′, reverse: 5′‐ GGAGACCCAGCAGCCTCAAA ‐3′) was used as a housekeeping gene. Real time quantitative polymerase chain reaction was then performed on a real‐time fluorescent quantitative PCR System.

### Flow Cytometric Analysis and Fluorescence Staining Assay

To measure intracellular ROS level, Raw 264.7 macrophages were incubated with H_2_S‐releasing scaffolds followed by treatment of 500 ng mL^−1^ LPS for 24 h. After removing the medium and washing the cells with PBS, serum‐free 1640 medium containing 0.1% DCFH‐DA was added to incubate cells for 30 min at 37 °C in dark. Next, cells were washed and collected to be analyzed using Fluorescence Activating Cell Sorter (FACS). Data were analyzed using FlowJo software.

The procedure of fluorescence staining assay can refer to the above steps. Fluorescent microscope was used to observe the generation of ROS within cells following the DAPI nuclear staining.

### Implantation of Band‐Aid Like AAB/BPB Patch

A total of 80 male Sprague‐Dawley (SD) rats (200 ± 20 g) were purchased from Guangdong Medical Laboratory Animal Center. The animal experiments were approved by the Animal Research Committee at Southern Medical University (LAEC‐076). Rats were randomly divided into four groups: sham, MI, MI+BPB, and MI+AAB/BPB. To establish MI rat mode, left anterior descending (LAD) coronary arteries of rats were ligated according to the reported protocol.^[^ ^98^ ^]^ Rats were first anesthetized with 2% (w/v) pentobarbital sodium (0.3 mL 100 g^−1^) by intraperitoneal injection. Next, the left anterior descending coronary artery was ligated with 8‐0 polypropylene suture at a position 2–3 mm away from its origin. Myocardial ischemia was determined by regional cyanosis on the front of the heart. The patch was implanted onto the infarcted area based on the ligation for rats. The implantation of AAB/BPB scaffold was performed as follows. First, 20 µL BPNSs solution (200 µg mL^−1^) was added around the albumin scaffold (10 mm × 10 mm, 60–70 µm) to prepare BPB before integrating with AAB. Next, AAB was prepared as described above and cut into 5 mm × 5 mm pieces, and then one piece was placed in the center of BPB to construct a band‐aid like cardiac patch (AAB/BPB). Following, the combined patch was placed on the left ventricular epicardial surface and irradiated with the 808‐nm diode laser (1.5 W cm^−2^ laser power fluxes, 60 s). At last, lateral chests of rats were immediately closed after near infrared irradiation.

### Immunofluorescence Staining of Macrophages in Myocardial Infarcted Area

The normal or infarcted hearts of Sham, MI, MI+BPB, and MI+AAB/BPB groups were excised on day 4 after MI, fixed in 4% (w/v) formaldehyde, and cut into paraffin sections. The heart sections were then rehydrated in gradient concentration of ethanol, and transparent by xylene. Next, the sections were permeabilized in 0.3% Triton X‐100 for 20 min and blocked with immunofluorescence staining blocking solution after antigen‐repairing with citrate antigen retrieval solution. After that, the heart sections were incubated with primary antibodies overnight at 4 °C for the detection of M1 macrophages (mouse anti‐iNOS, Abcam) and M2 macrophages (rabbit anti‐CD206, CST). Thereafter, the sections were washed with PBS and incubated with Cy3‐labeled secondary antibodies (goat antirabbit IgG, Bioworld) and 488‐labeled secondary antibodies (goat antimouse IgG, Bioword) for 60 min. Finally, DAPI staining for cell nuclei were carried out. All the heart sections were visualized using a confocal laser‐scanning microscope (Nikon, Tokyo, Japan) and the distribution of macrophages was analyzed by the Image J software.

### Echocardiographic Assessment of Left Ventricular Function

The left ventricular functions of the rats were assessed by an ultrasound system (Visual Sonics VEVO2100) on 7, 14, 21, and 28 days after surgery. The ejection fraction (EF), end‐diastolic volume (EDV), end‐systolic volume (ESV), and fractional shortening (FS) of rat left ventricle (LV) were examined as indicators of the LV systolic function and remodeling situation.

### Masson's Trichrome Staining

To evaluate the level of myocardial fibrosis at 4 weeks after patch implantation, rats in each group were anesthetized as above and the hearts were excised, fixed in 4% (w/v) formaldehyde, and cut into paraffin sections. Next, the sections from the ligation point to the apex of the heart were stained by Masson's trichrome staining, and then the fibrosis area were calculated by the Image J software.

### Statistical Analysis

All results are expressed as mean ± standard deviation (at least *n* = 3). Student's *t*‐test and One‐way ANOVA were used to determine the statistical significance. The significant levels were set as *p < 0.05, **p < 0.01, ***p < 0.001, or ns, nonsignificant.

## Conflict of Interest

The authors declare no conflict of interest.

## Supporting information

Supporting Information

Supplemental Movie 1

Supplemental Movie 2

## Data Availability

The data that support the findings of this study are available from the corresponding author upon reasonable request.
