# Supplementary material for: Construction of a Band‐Aid Like Cardiac Patch for Myocardial Infarction with Controllable H2S Release
Source: Adv Sci (Weinh). 2022 Oct 26;9(35):2204509. doi: 10.1002/advs.202204509 (PMC9762300; doi:10.1002/advs.202204509)
Supplement: Supplementary file 1 — Supporting Information [file ADVS-9-2204509-s001.pdf]

ADVANCED SCIENCE
Open Access

Supporting Information

for *Adv. Sci.*, DOI 10.1002/advs.202204509

Construction of a Band-Aid Like Cardiac Patch for Myocardial Infarction with Controllable H_2S Release

Weirun Li, Peier Chen, Yuxuan Pan, Ling Lu, Xiaodong Ning, Jiamin Liu, Jintao Wei, Minsheng Chen, Peng Zhao and Caiwen Ou**

Supporting Information

Construction of a Band-aid like Cardiac Patch for Myocardial Infarction with Controllable H_2S Release

Weirun Li[1,6], Peier Chen[3,6], Yuxuan Pan[1], Ling Lu[4], Xiaodong Ning[1], Jiamin Liu[4], Jintao Wei[1], Minsheng Chen[3], Peng Zhao[4,5] and Caiwen Ou[1,2]**

1. Affiliated Dongguan Hospital, Southern Medical University (Dongguan People's Hospital), Dongguan 523058, China
2. Guangdong Provincial Key Laboratory of Shock and Microcirculation, Guangzhou 510515, China;
3. Department of Cardiology, Laboratory of Heart Center, Heart Center, Zhujiang Hospital, Southern Medical University, Guangzhou 510280, China
4. NMPA Key Laboratory for Research and Evaluation of Drug Metabolism, Guangdong Provincial Key Laboratory of New Drug Screening, School of Pharmaceutical Sciences, Southern Medical University, Guangzhou 510515, China
5. Guangdong Provincial Key Laboratory of Cardiac Function and Microcirculation, Southern Medical University, Guangzhou 510515, China
6. These authors contributed equally to this work.

Conflict of Interest: The authors declare no conflict of interest

*Correspondence: oucaiwen@smu.edu.cn (Caiwen Ou), smuzp@smu.edu.cn (Peng Zhao)

Supplementary materials and methods

Experimental Reagents and Antibodies

Sodium alginate (M/G = 1:2, AR), N-phenyl-p-phenylenediamine (AR), reductive glutathione (GSH, BC), sodium periodate ($NaIO_4$, AR), β-mercaptoethanol (biotech grade), 2,2,2-Trifluoroethanol (TFE, LR) and bovine serum albumin (BSA, biotech grade) were purchased from Macklin (Shanghai, China). Dimethyl sulfoxide (DMSO, AR) and 2-aminopyridine-5-thiocarboxamide (ATPC, AR) was purchased from Sigma-Aldrich (USA). Absolute ethanol (AR) and ethylene glycol (AR) were purchased from Aladdin (Shanghai, China). Deionized water was provided by Southern Medical University. Dulbecco's Modified Eagle Medium (DMEM, high glucose, Gibco, USA), RPMI 1640 Medium (Gibco, USA), fetal bovine serum (FBS) and 1% penicillin-streptomycin (Gibco, USA) were used to culture cells.

Antibodies specific to CD206 (Cell Signaling technology, #24595), Arg-1 (Affinity, DF6657), iNOS (Abcam, ab210823), IL-18 (Abcam, ab207323), Connexin 43 (Servicebio, GB11234), α-actinin2 (Servicebio, GB12555), CD31 (Servicebio, GB13063), α-smooth muscle actin (Servicebio, GB111364), β-actinin (Bioworld, AP0060).

Preparation of Black Phosphorus Nanosheets

The black phosphorus nanosheets (BPNSs) were prepared by liquid-phase exfoliation.[1, 2] In brief, 50 mL of deionized water was added to a flask with a double-lane valve. One entrance of valve was inflated with pure nitrogen gas as needed and the other entrance was linked to a vacuum pump. Firstly, the air in the flask was exhausted as far as possible through suction under negative pressure. Fifteen minutes later the pipe between the flask and vacuum pump was closed and nitrogen gas was then filled in flask, followed by sloshing deionized water in the flask for 10 min. Deoxygenated water was obtained after repeating the above steps 5 times. Secondly, 20 mg of black phosphorus crystal powder (purchased from XFNANO, Jiangsu, China) was added to flask quickly and dispersed in deoxygenated water. The mixed solution was then homogenized through sonicate in ice bath for 7 h. The stock

solution was finally obtained after centrifugation at 2,000 rpm for 10 min and stored under 4 °C for further use. Before use, the stock solution was centrifuged at 12,000 rpm for 15 min.

Cell Viability Assay

Cell viability assays were performed on the BSA scaffold, BSA scaffold with BPNSs with or without near infrared irradiation and AAB with Live/Dead staining. A Calcein/PI Cell Viability/Cytotoxicity Assay Kit (Beyotime, C2015M) was used to assess the viability of primary cardiomyocytes grown on the BSA scaffold or co-cultured with AAB. We first evaluated the cytotoxicity of the thermal energy generated by BPNSs on cardiomyocytes seeded onto the scaffold after irradiated with 808 lasers (90 s, 1.5 W cm^{-2}). The cardiomyocytes were cultured on scaffold for 5 days, and then the old medium was removed before adding BPNSs solution (20 μL, 200 μg mL^{-1}) to the albumin scaffold. After irradiation, cardiomyocytes were incubated for approximately 30 min with AM (2 μL mL^{-1}) and PI (3 μL mL^{-1}) in pure DMEM. The cytotoxicity of AAB on cardiomyocytes was also evaluated with Live/dead staining after coculture for 48 h. Live and dead cells were visualized under a fluorescence microscope (Nikon multipurpose microscope, Japan) using a 5× objective lens. The images were processed by ImageJ software.

Cell Culture

Mouse monocyte macrophages of cell line Raw 264.7 were purchased from American Type Culture collection and cultivated in RPMI 1640 medium containing 10% fetal bovine serum, 100-unit mL^{-1} penicillin and 100 μg mL^{-1} streptomycin in a controlled incubator at 37 °C with an atmosphere of 5% CO_2. The human umbilical vein endothelial cells (HUVECs) used in our study were purchased from the cell bank of the Chinese Academy of Sciences (Shanghai, China) and cultured in DMEM (10% FBS and 1% penicillin-streptomycin).

Primary cardiomyocytes were obtained from new-born Sprague Dawley rats as previously described.[3] Briefly speaking, after disinfecting suckling rat with alcohol, the heart was harvested and then put in bottle containing trypsin for digestion at 4 °C overnight. DMEM medium that contain 10% FBS was added to terminate digestion

for 5 min at 37 °C the next day. After that, the old medium was replaced with an equal volume of collagenase (0.1%) to further digest tissues at rotating speed of 200 rpm for 5 min at 37 °C, following which the supernatant was collected. The isolated cells were obtained after centrifugation at 800 rpm for 3 min and then cultured in cell culture dish for 2 h. At last, non-adherent primary cardiomyocytes were transferred to other cell culture dishes or immediately seeded on the scaffolds (15 mm × 15 mm, 1×10^6 cells) for the next experiments. The cell-seeded samples were cultivated in DMEM culture medium containing 10% FBS at 37 °C and 5% CO2. The culture medium was changed daily.

Migration Assay

The cell scratch test and Transwell assay were applied to assess the migration of HUVECs, which can indirectly explain the angiogenesis.[4, 5] Firstly, 5×10^5 cells/well were planted in a 6-well plate and cultured to confluence for the wound healing assay. The monolayer of HUVECs was scraped with a 200 μL pipette tip and rinsed with PBS to remove breakaway cells. AAB and BPB (BPNSs, 200 ng mL^{-1}) were then added to each well. Images were taken using an inverted phase microscope at 0, 6, 12 and 24 h after scratch. The migration area to evaluate wound healing was calculated as follows: migration area (%) = $(A_0–A_n)/A0 \times 100\%$, where A_0 represents the initial wound area and A_n represents the wound area at the time of measurement. The Transwell assay was performed by using Transwell cell culture inserts (8 μm pore size; FALCON, USA) in 24-well plates. HUVECs (about 3×10^4 cells/well) were suspended in a low-serum (1% FBS) medium and cultivated in the upper chamber. The lower chamber contained 700 μL of complete medium supplemented with 20% FBS. The non-migrating cells in the upper chambers were gently removed after intervention 24 h, and the cells on the outside of the filters were counted after staining with 0.1% crystal violet and photographed by an optical microscope.

Supplementary figures

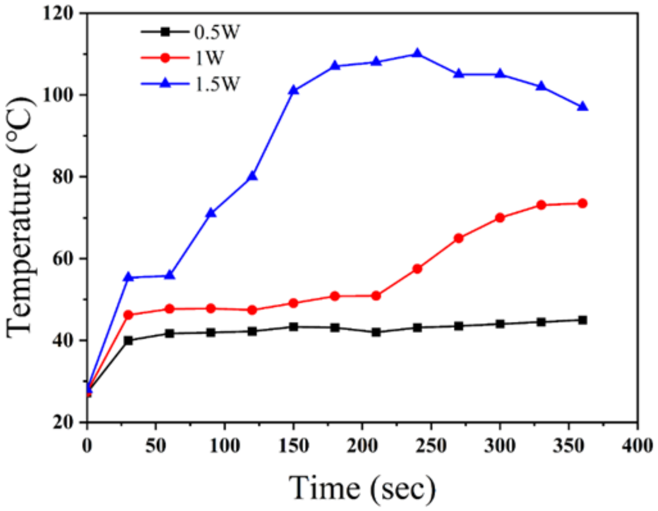

Figure S1. Photothermal heating curves of albumin fiber scaffolds with the addition of BPNPs (200 μg/ml, 20μl) under irradiation with an 808 nm laser in different laser power flux (0.5 Wcm^{-2}, 1.0 Wcm^{-2} and 1.5 Wcm^{-2}).

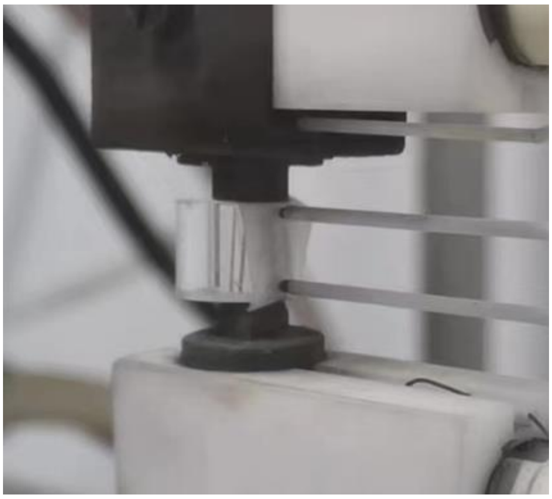

Figure S2. Picture of measurement of conductivity of BPB scaffold.

Table S1. Oxidation degree of sodium alginate.

Molar ratio (%)	Degree of oxidation (%)
0	0
50	43.6±3.0
80	64.4±4.7
100	69.3±3.4

Molar ratio is the ratio of NaIO4 and alginate units. The degree of oxidation (DO)

is examined by hydroxylamine hydrochloride-potentiometric titration (DO = (1/2 Aldehyde)/Alginate units).

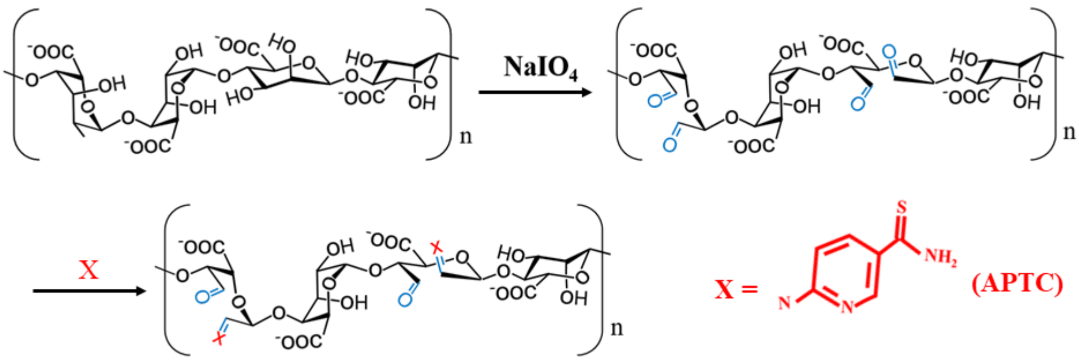

Figure S3. Chemical synthesis of ALG-APTC. APTC was linked onto partially oxidized sodium alginate based on the Schiff base reaction between amino and aldehyde to prepared a H_2S-releasing copolymer.

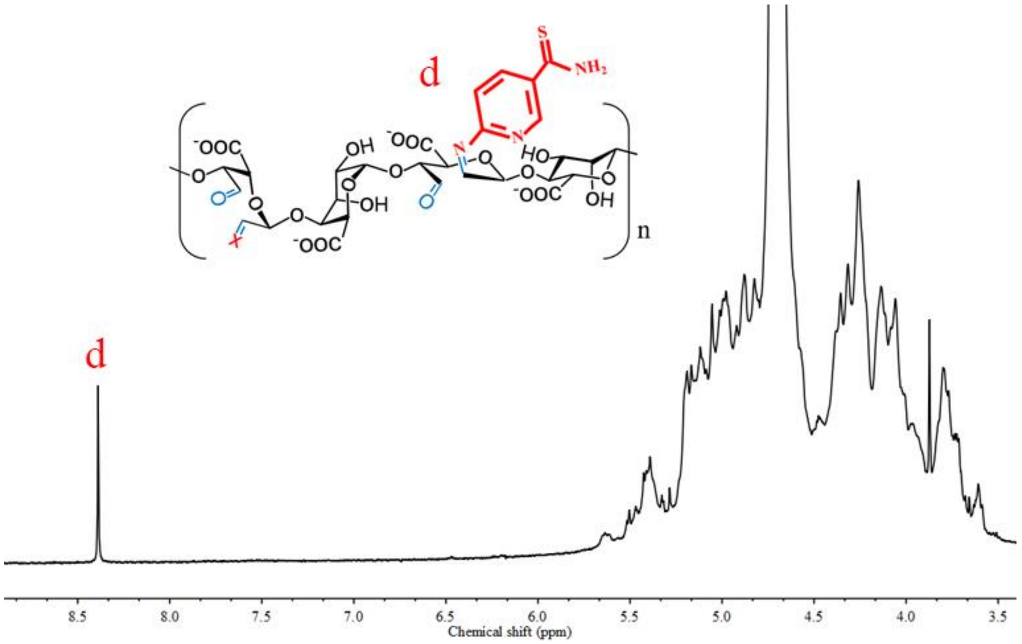

Figure S4. Characterization of APTC-ALG (AA). The photograph shown above is the ^{1}H NMR spectrum of AA and the illustration presented in the upper left-hand corner is the chemical structural formula of AA.

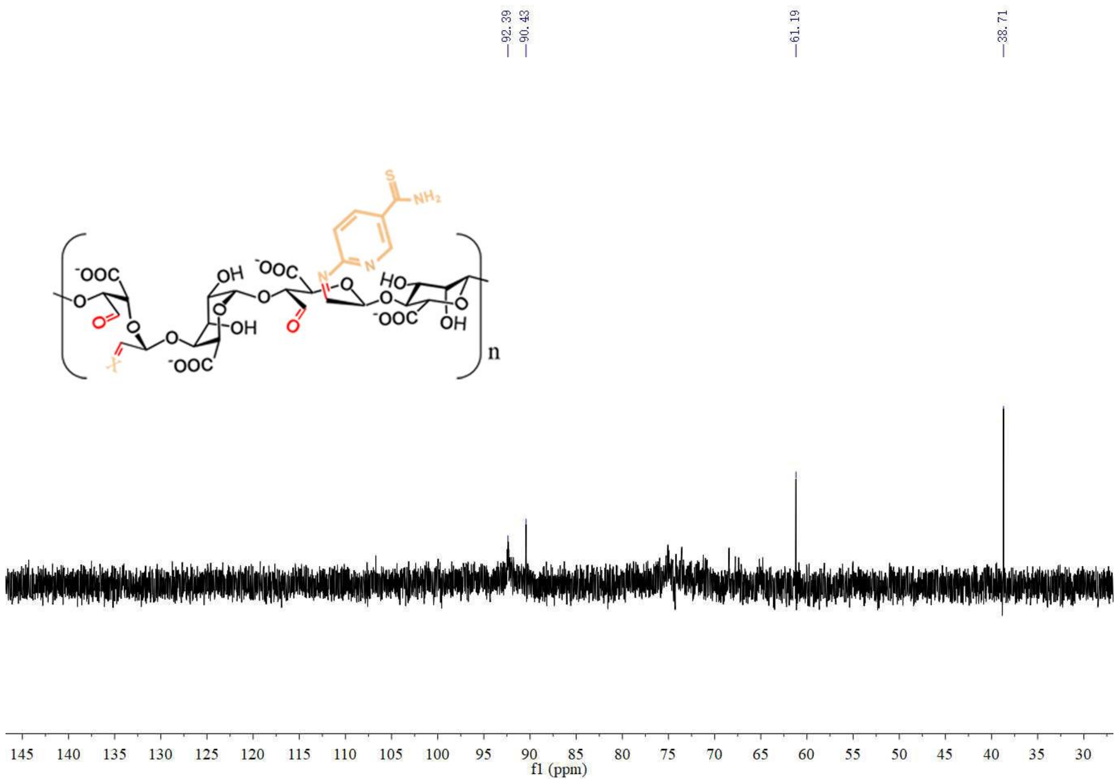

Figure S5. C-NMR spectrum of AA. Peaks above 90 ppm are assigned to carbon-carbon double bond (sp~2 hybrid C) which existed only in APTC, while peaks below 60 ppm are attributed to the single bonds between carbons (sp~3 hybrid) in ALG.

Table S2. Elemental composition of ALG-APTC.

Element	C	N	H	S
Weight Percentage (%)	26.85	0.64	5.023	2.683

Elemental composition of ALG-APTC measured by organic element analysis and detection. The mass fraction of APTC is calculated as 2.33wt% based on the content of N.

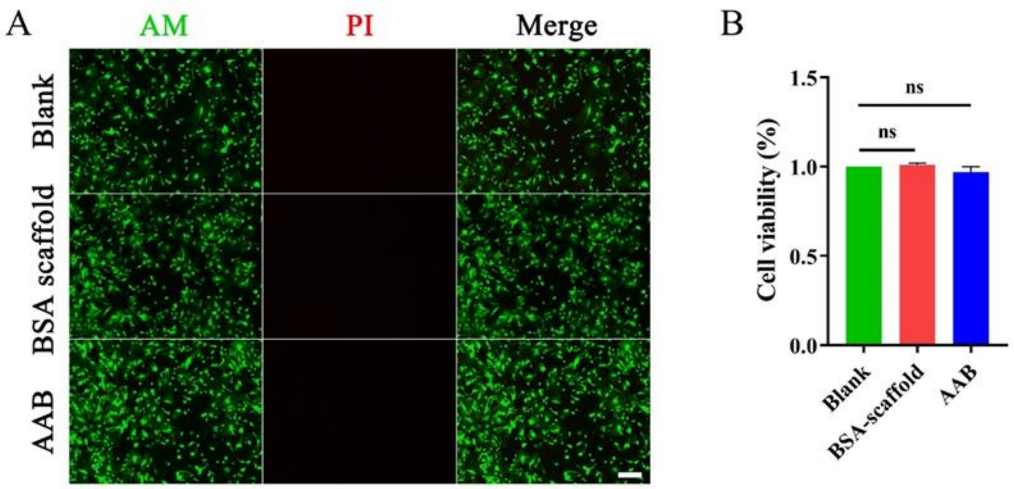

Figure S6. Evaluation of the cytotoxicity of AAB on cardiomyocytes. Representative images of neonatal rat primary cardiomyocytes stained with Calcein-AM (green) and PI (red). Scar bar: 150 μm. Quantified data are presented as means ± SD, and significance was evaluated via Student's *t* test. *p <0.05; ns, nonsignificant.

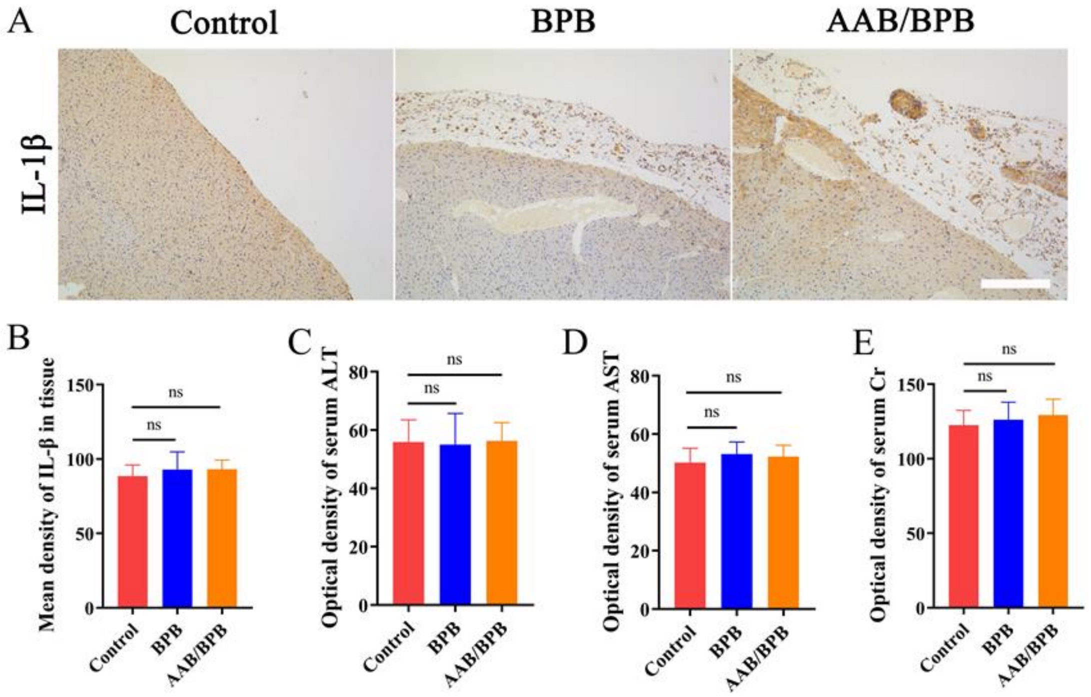

Figure S7. Cytotoxicity and biocompatibility of AAB/BPB patch. (A) Immunohistochemical staining of IL-1β on day28. Scale bar: 200 μm. (B) Quantitative analysis of IL-1β expression in (A) (n = 4). (C-E) The detection of blood biochemistry indexes (ALT, AST, Cr) by ELISA (n = 4). Quantified data are presented as means ± SD, and significance was evaluated via One-way ANOVA. ns, nonsignificant.

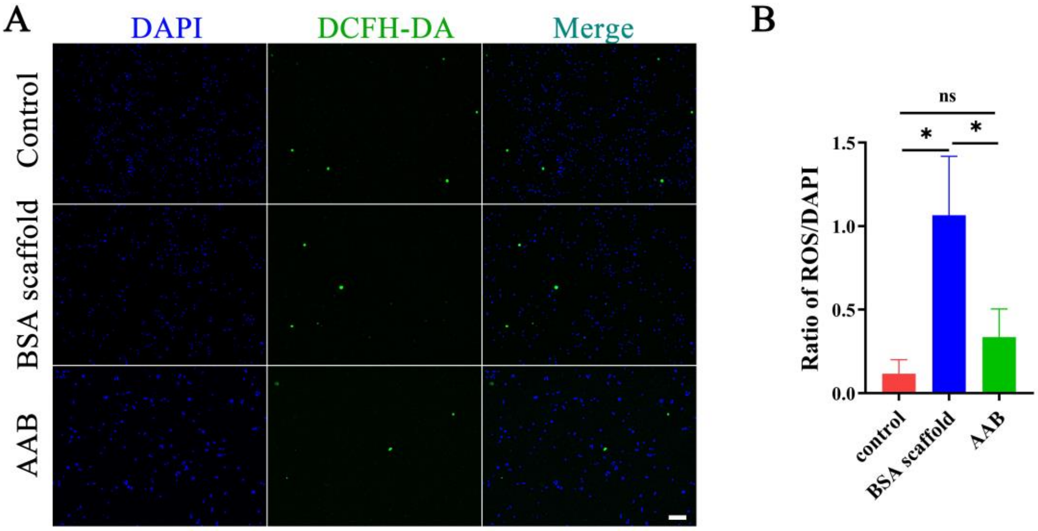

Figure S8. Detection of intracellular reactive oxygen species. (A) Representative images of DCFH-DA fluorescent staining of ROS in macrophages under different treatments for 24 h. Scale bar: 100 μm. (B) Quantified data of relative generation of ROS (n = 3). Quantified data are presented as means ± SD, and significance was evaluated via Student's *t* test. *p <0.05; ns, nonsignificant.

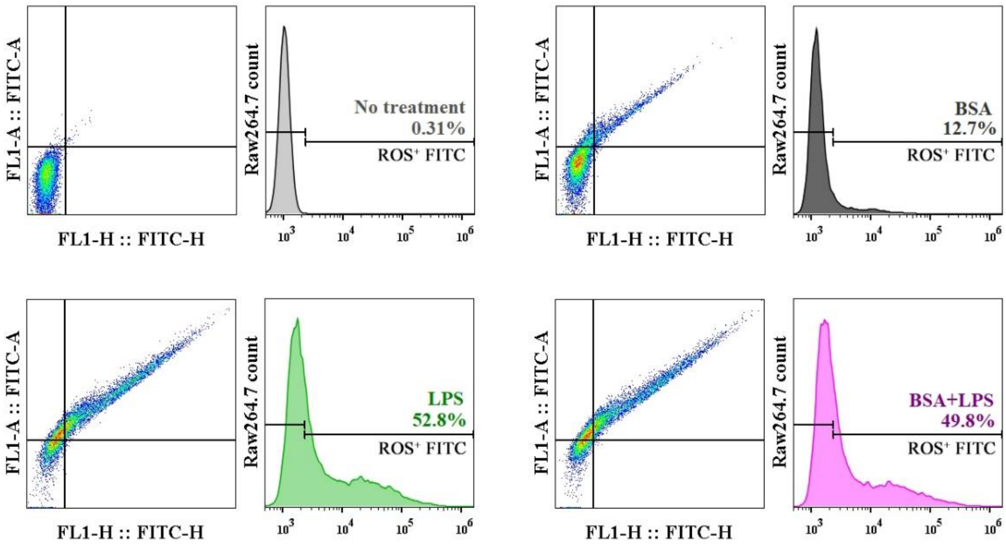

Figure S9. Detection of intracellular reactive oxygen species. Flow cytometric analysis of DCFH-DA fluorescent labeled macrophages under different treatments for 24 h.

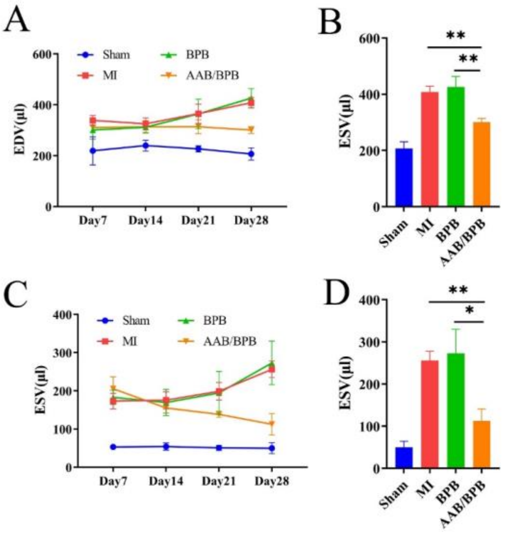

Figure S10. The cardiac functions and left ventricular remodeling of rats 28 days after operation were assessed. (A) and (C) Changes of EDV and ESV measured by echocardiography at 7, 14, 21 and 28 days after different treatments. (B) and (D) Changes of EDV and ESV at 28 days after MI. Quantified data are presented as means ± SD, and significance was evaluated via Student's *t* test. *p <0.05, **p <0.01.

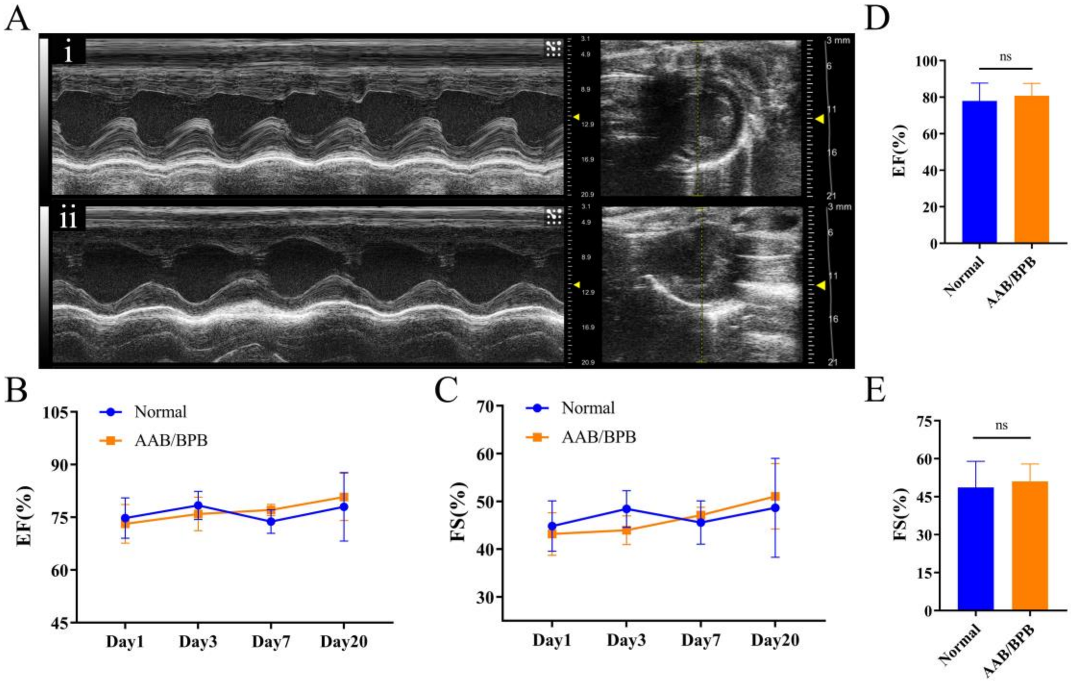

Figure S11. Evaluation of AAB/BPB patch on normal cardiac function. (A) Representative echocardiography (ECHO) images of normal (i) and AAB/BPB group (ii) on day20. Echocardiographic assessments of left ventricular EF (B) and FS (C)

were conducted on day1, 3, 7 and 20 (n=3). Statistical analysis of EF (D) and FS (E) on day 20 (n = 3). Quantified data are presented as means ± SD, and significance was evaluated via Student's *t* test. ns, nonsignificant.

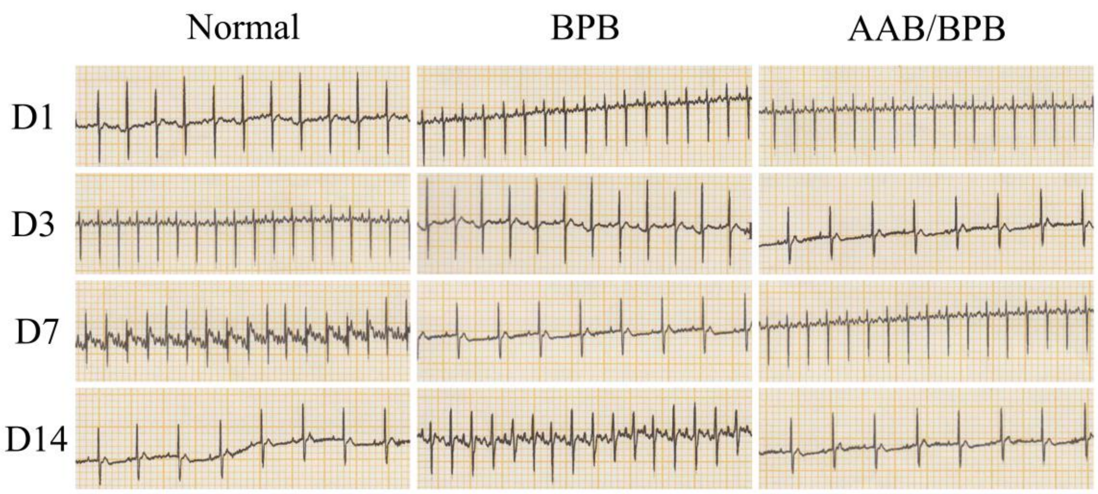

Figure S12. ECG detection of non-treated or treated with BPB or AAB/BPB rats.

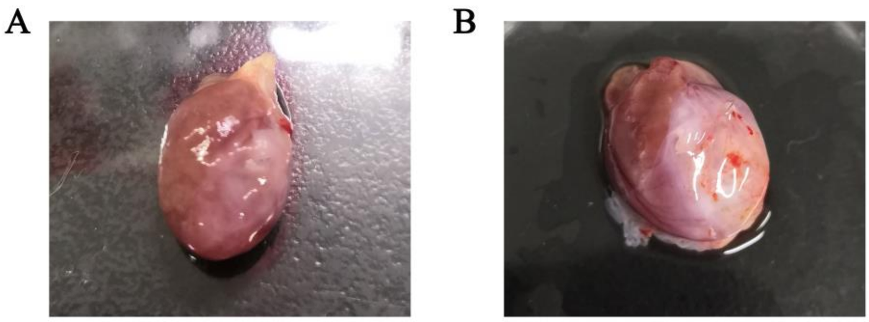

Figure S13. Picture of attachment of BPB scaffold (A) and AAB/BPB patch (B) to the tissues on 4 weeks after implantation.

Movie S1. The shaking of electrospun scaffold can be observed through a microscope under the pull of beating cardiomyocytes.

Movie S2. Cardiomyocytes' pulsation can be observed through a fluorescence microscope after staining with Calcein-AM (green) and PI (red).

References

1. He, J.; Chen, G.; Zhao, P.; Ou, C., *Nano Res.* **2021,** *14* (11), 3988-3998.
2. Qiu, M.; Wang, D.; Liang, W.; Liu, L.; Zhang, Y.; Chen, X.; Sang, D. K.; Xing, C.; Li, Z.; Dong, B.; Xing, F.; Fan, D.; Bao, S.; Zhang, H.; Cao, Y.,. *Proc. Natl. Acad. Sci. USA* **2018,** *115*, 501-506.
3. Hongyu; Sun; Shuanghong; Lü; Xiao-Xia; Jiang; Xia; Li; Hong; Biomaterials, L. J., *Biomaterials* **2015**, *55*, 84-95.
4. Liu, L.; Liu, Y.; Feng, C.; Chang, J.; Fu, R.; Wu, T.; Yu, F.; Wang, X.; Xia, L.; Wu, C. J. B., *Biomaterials* **2018,** *192*, 523-536.
5. Liu, S.; Chen, J.; Shi, J.; Zhou, W.; Wang, L.; Fang, W.; Zhong, Y.; Chen, X.; Chen, Y.; Sabri, A.; Liu, S., *Basic Res. Cardiol.* **2020,** *115*, 22.
